# 

**Published:** 1893-11

**Authors:** 


### CONTENTS FOR NOVEMBER, 1893.

ORIGINAL COMMUNICATIONS. PAGE.

Paranoia, with Delusions of Change in Sex. By C. B. Burr, M. D. .... 193
Statistics of Infectious Diseases. By Franklin C. Gram, M. D. .... 197
Causes and Modes of Communication of Contagious and Infectious Diseases. By Edward Clark, M. D. .... 201
What the Newer Therapeutic Procedures have Done for Neurology. By William C. Krauss, M. D. .... 212

CLINICAL LECTURE.

Clinical Memoranda from the Surgical Clinic at the Sisters' of Charity Hospital. By Herman Mynter, M. D. .... 221

EDITORIAL.

State Examination for License .... 227
The Resignation of Dr. Rochester .... 230
Topics of the Month .... 231

PERSONAL.

Dr. Roswell Park .... 236

OBITUARY.

Professor John Michael Maisch, M. D. .... 236
Dr. Walter Vought—Dr. Allen Augustus Stevens .... 237

Society Meetings .... 238
Academy of Medicine Notes .... 238
Book Reviews .... 239
Books Received .... 254
Miscellany .... 255

